# A Case of Shamblin III Carotid Body Tumor

**DOI:** 10.7759/cureus.97224

**Published:** 2025-11-19

**Authors:** Soman N Iqbal, Kishwar Ali, Sahibzada Muhammad A Gondal, Zulkiffil Mubarak

**Affiliations:** 1 Medicine, Foundation University Islamabad, Islamabad, PAK; 2 Vascular Surgery, Fauji Foundation Hospital, Rawalpindi, PAK; 3 General Surgery, Fauji Foundation Hospital, Rawalpindi, PAK

**Keywords:** carotid artery surgery, carotid body tumor, head and neck tumors, neck lump, shamblin type iii

## Abstract

Carotid body tumors (CBTs) arise from the chemoreceptor cells located at the base of the carotid arteries. Due to their proximity to crucial structures, CBTs are diagnostic and therapeutic challenges. Most of these tumors are benign; however, a small proportion of these malignant tumors have a good survival rate. Shamblin classified these tumors based on their local spread into three stages. This case report describes the complexities of a Shamblin III CBT. A woman in her mid-40s presented with a 13-year history of a slow-growing, painless lump on the left side of her neck, which had gone unnoticed until it reached a size of considerable magnitude. She did not have any concerning symptoms, such as fever, sore throat, weight loss, or night sweats. There were no other red flag symptoms, and her past medical history was unremarkable. There was no history suggestive of exposure to tuberculosis. During the examination, the patient was vitally stable and presented with an obvious, irregularly shaped, pulsatile, firm, and smooth mass on the left lateral portion of the neck that measured 6×3 cm. The mass also had an audible bruit. The other parts of the neck examination were also unremarkable, and there was no lymphadenopathy. Differential diagnosis in this case would include lipoma, cystic lesion, malignancy in the head and neck, and CBT.

Baseline investigations, along with serological tests, were normal. As per computed tomography (CT) angiography findings, a 5.5×6.3×8.5 cm mass was found in the left carotid space. This mass was enhancing, splitting the internal and external carotid arteries on both sides and completely enclosing the bifurcation of the carotids along with their tributaries. This was classified as a Shamblin III CBT.

The size and location of the encapsulating mass warranted a definitive surgical approach. The procedure was carried out as a planned excision under general anesthesia. After general risk counseling and the acquisition of informed consent, the anterior border of the sternocleidomastoid muscle was incised. Once the carotid sheath was exposed and the internal jugular vein retracted, the mass was visible. The encapsulating mass was closely surrounding the common, internal, and external carotid arteries. Care was taken during the resection of the mass to preserve the surrounding structures. The mass was completely resected along with the distal end of the external carotid artery. A Javid shunt was placed, and then the shunt was removed. After hemostasis, the wound was reapproximated, and the mass was sent for complete pathological review. The patient remained in the hospital for four days and showed uneventful progression. She was discharged on the fourth postoperative day.

Considering a broad differential diagnosis for painless neck masses is important. For Shamblin III CBTs, imaging is critical in diagnosis, formulating a preoperative plan, and understanding the extent of difficulties posed by the excision of these vascular tumors. Surgical excision, which is highly challenging, is still the best approach to take to treat these tumors.

## Introduction

Located at the base of the carotid arteries, carotid body tumors (CBTs) present a clinical dilemma for surgeons. These rare tumors, arising from chemoreceptor cells, pose a diagnostic and therapeutic challenge due to their close proximity to vital vessels and, of course, a potential for malignancy [1]. Surgical resection is the mainstay of treatment, but navigating the intricate vascular architecture involves risks of cranial nerve palsy and also vascular complications [2]. Adding to the complexity of the surgery, the preoperative diagnosis can be troublesome; often, it is obscured by various imaging presentations and a potential misidentification due to structures such as lymph nodes or schwannomas [3].

The majority of these tumors are benign, and only about 6% are malignant. Even with the malignant types, when regional lymph nodes are found, the reported five-year survival rate is about 60% [4,5].

Shamblin has described three stages of these tumors based on local spread. Stage I involves small tumors that are easily dissectible, and stage II involves the vessels partially, while stage 3 completely surrounds the carotid bifurcation [6]. This specific case report delves into the intricate case of a Shamblin III CBT.

Radiological investigations included contrast-enhanced computed tomography (CT) scans and magnetic resonance imaging (MRI). T2-weighted MR images show hyperintense lesions [7]. Due to their vascularity, they have what's called a "salt-and-pepper" pattern on MRI. This is due to the high-velocity flow voids (black dots) and slow flow (white dots) [8]. Angiography is also sometimes used in patients undergoing resection; the scan demonstrates splaying of the internal and external carotid arteries, which is also known as the Lyre sign [9].

Through a comprehensive analysis of this unique case, the aim is to illuminate the diagnostic shadows cast by CBTs and to refine management protocols. This is to ultimately improve patient outcomes.

## Case presentation

This is the case of a woman in her mid-40s who presented with a 13-year history of a slow-growing, painless lump on the left side of her neck. Initially, the lump progressed unnoticed until it reached a significant size and became visibly prominent. This was not associated with fever, sore throat, weight loss, night sweats, or any history of trauma. Her past medical history was unremarkable, with no prior hospital admissions or medication use. There was also no family history of tuberculosis or similar neck lesions.

**Figure 1:**
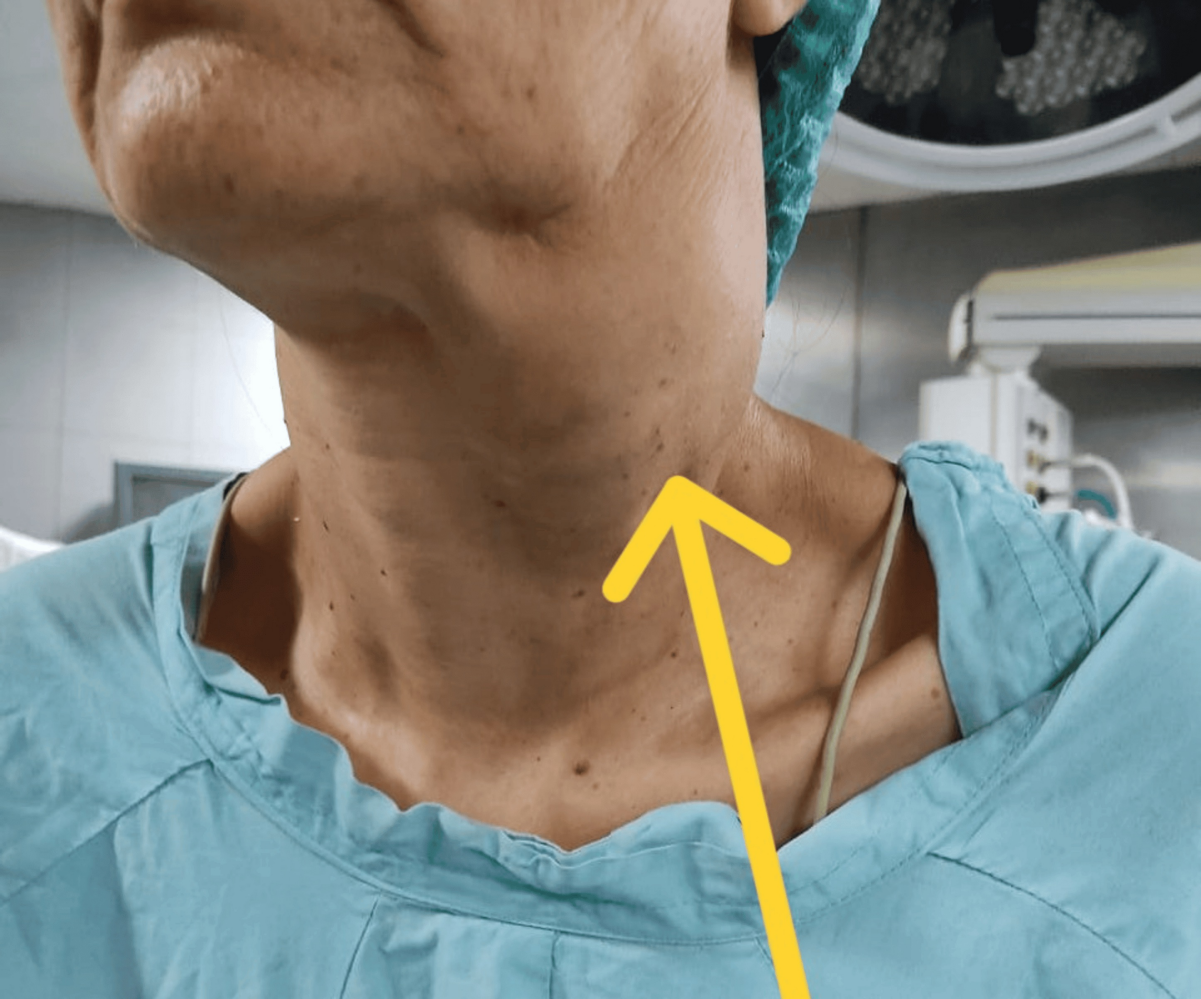
An obvious irregular mass in the left lateral aspect of the neck

On general physical examination, she was a stable and well-oriented woman with normal vital signs, and on local examination, there was a 6×3 cm irregular mass in the left lateral aspect of her neck (Figure 1). It was pulsatile and firm and had a smooth surface. It exhibited limited vertical mobility but was mobile horizontally. A prominent bruit was audible on auscultation over the mass. The rest of the examination of the ears, oral cavity, thyroid, and lymph nodes was unremarkable.

Investigations

Laboratory investigations included baseline complete blood count, renal function tests, liver function tests, electrolyte levels, coagulation profile, and serology, which were all within normal limits. Echocardiography was also normal. Further imaging with CT angiography revealed an intensely enhancing 5.5×6.3×8.5 cm mass in the left carotid space, splaying the internal and external carotid arteries with complete encasement of the carotid bifurcation and its tributaries. This was consistent with Shamblin classification type III.

Differential diagnoses

Initially, the neck mass raised several diagnostic possibilities. Given its slow growth and painless nature, benign causes like lipoma or cystic lesions were initially also considered. The mass's location in the lateral neck and its pulsatile character narrowed the options down a bit. Branchial cleft cysts are also a possibility; they arise from remnant embryonic tissue in this area. However, the absence of skin changes or drainage made this less likely. Reactive lymphadenopathy caused by chronic infections was also a possibility, but the lack of associated symptoms, lab workup, and normal lymph node examination made this unlikely. The combination of pulsatile mass, atypical location, and negative evaluation for common benign and inflammatory causes ultimately focused attention on more serious possibilities, including metastatic head and neck cancers. The CT angiogram findings of intense enhancement and splaying of the carotid arteries strongly suggested a CBT, which ultimately proved to be the accurate diagnosis after malignancy was ruled out on histopathology.

Management

**Figure 2:**
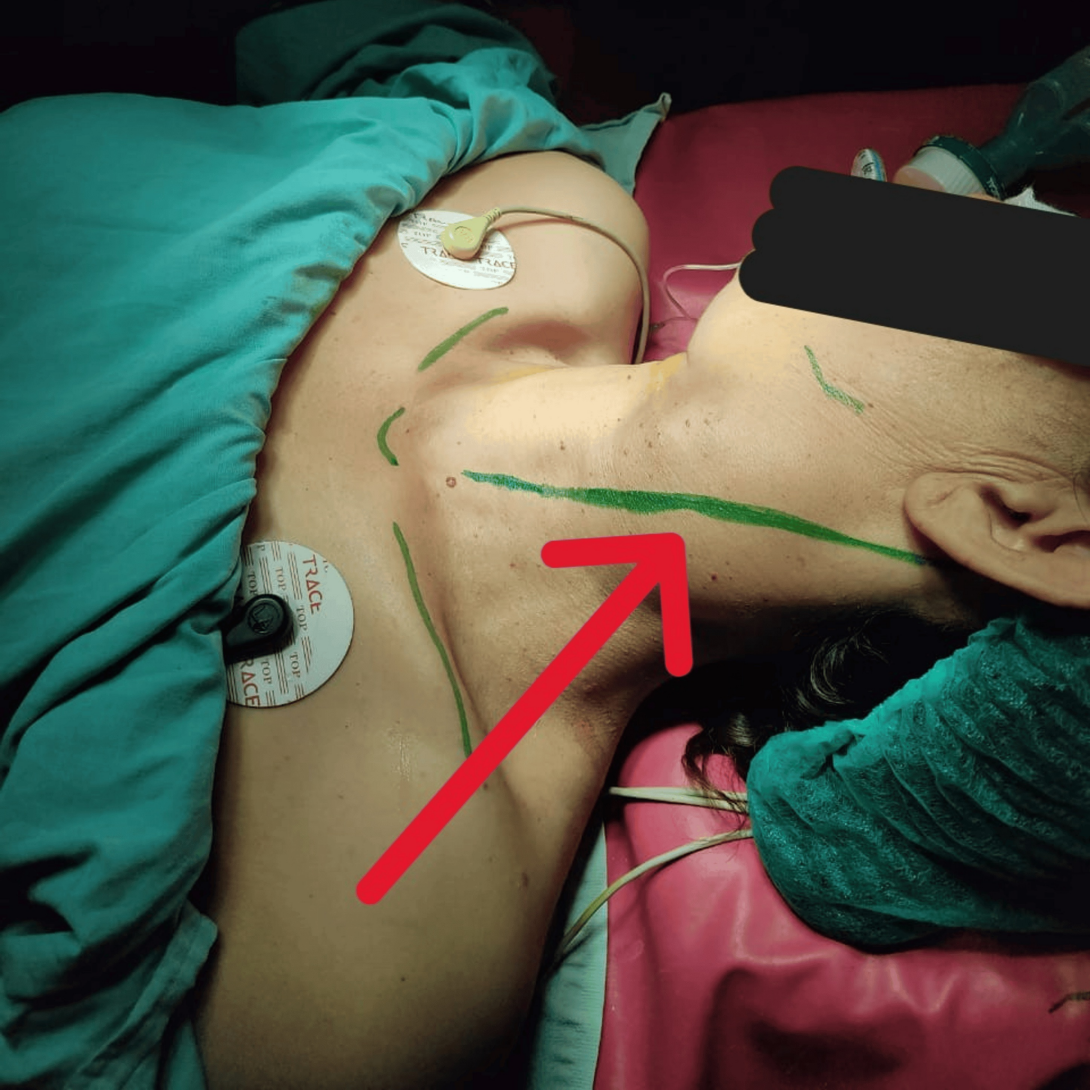
Marking of incision along the anterior border of the sternocleidomastoid muscle

**Figure 3:**
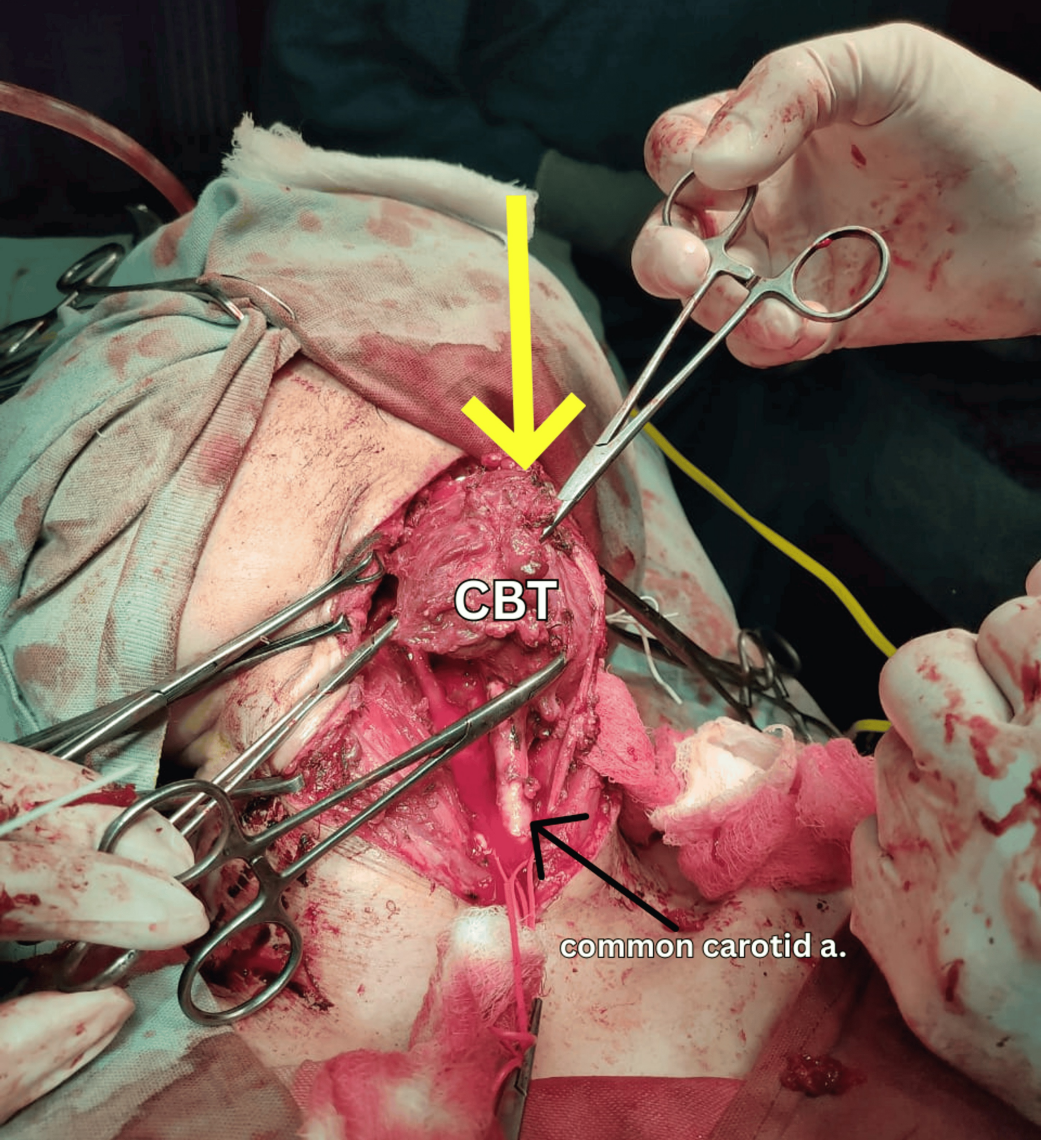
Intraoperative view demonstrating the sub-adventitial dissection (peeling) of the CBT off the common carotid artery towards the bifurcation CBT: carotid body tumor

**Figure 4:**
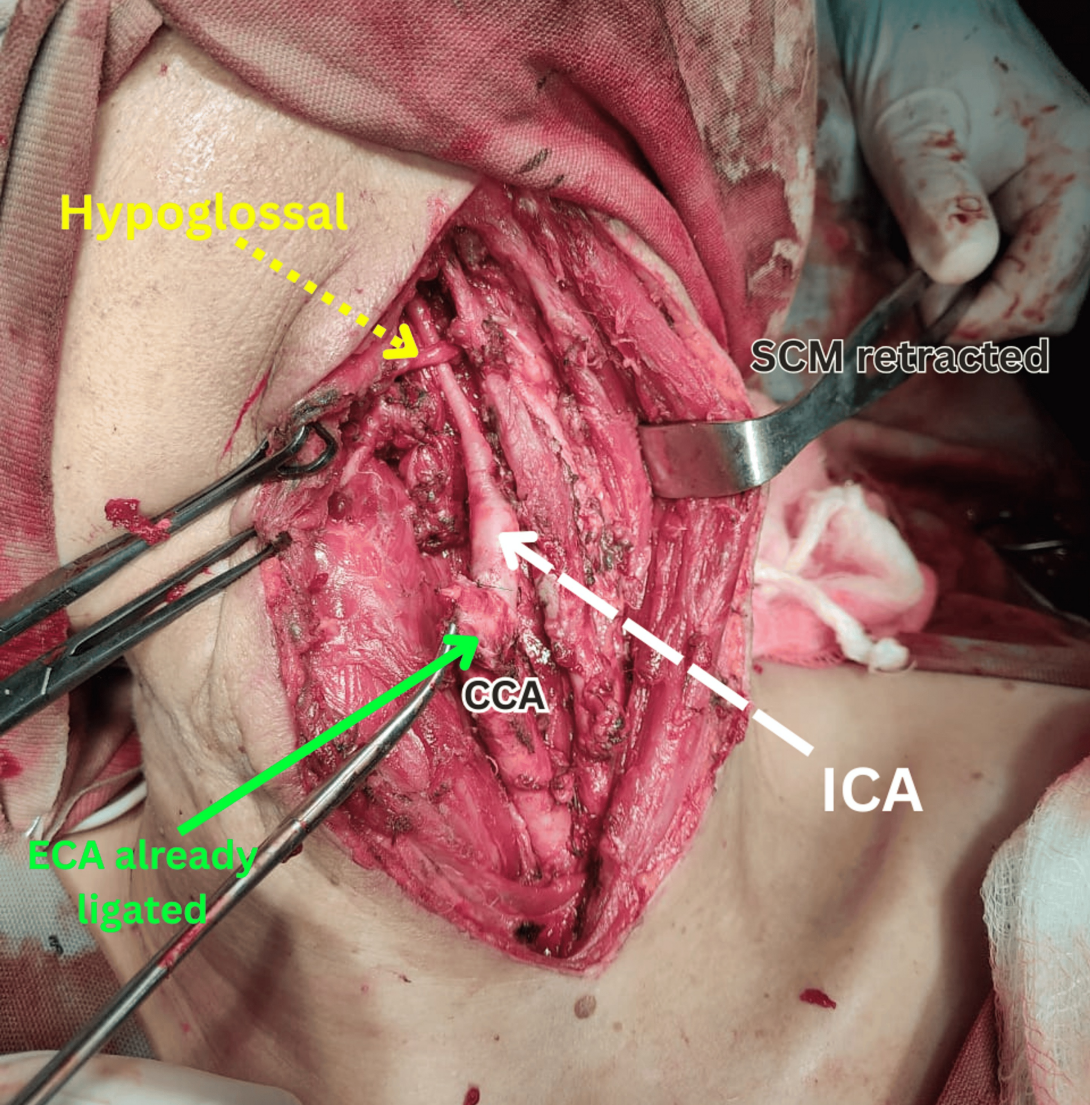
Intraoperative view after the complete excision of the CBT: ECA already ligated and ICA and hypoglossal nerve preserved CBT: carotid body; CCA: common carotid artery; ECA: external carotid artery; ICA: internal carotid artery; SCM: sternocleidomastoid muscle

**Figure 5:**
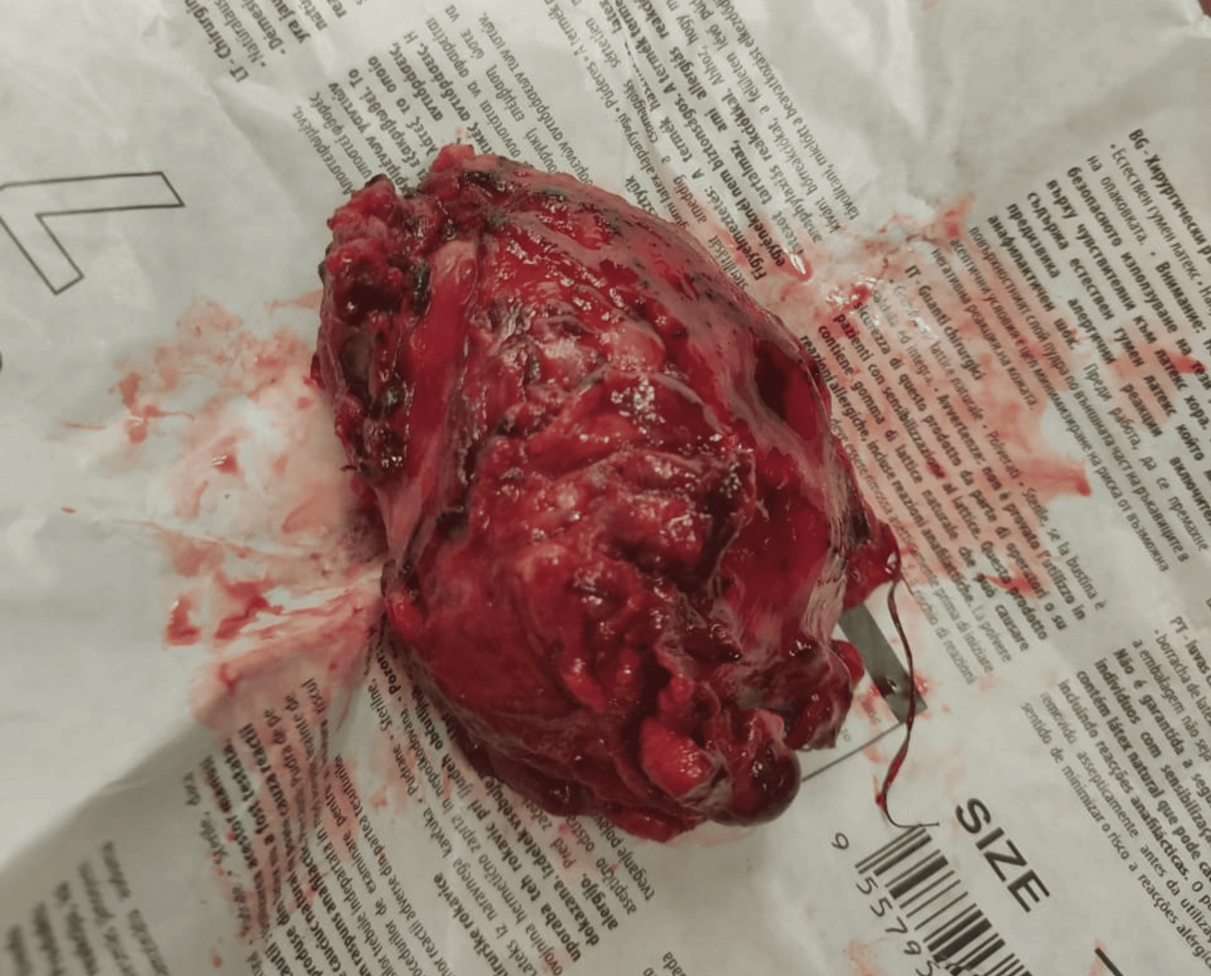
Tumor after complete resection

Due to the size and encasement of the tumor, surgical resection was chosen as the definitive treatment. Informed consent was obtained, and a thorough risk counselling was carried out, including potential complications like cranial nerve injuries and stroke. Under general anesthesia and with the head elevated slightly, a curved incision was made along the anterior border of the sternocleidomastoid muscle (Figure 2). After careful dissection, the carotid sheath was opened and the internal jugular vein retracted. The tumor, closely surrounding the common, internal, and external carotid arteries, was meticulously dissected using a combination of cranio-caudal and caudo-cranial approaches (Figure 3). First, the common carotid artery was controlled, followed by the identification and ligation of the external carotid artery (Figure 4). The tumor was then painstakingly separated from the internal carotid artery, critical for maintaining blood flow. Both the distal end of the external carotid artery and the entire tumor were ultimately ligated and excised (Figure 5). A Javid shunt, pre-emptively placed to maintain cerebral perfusion in case of unforeseen internal carotid artery compromise, remained unused. Following meticulous hemostasis, a drain was placed and the incision closed securely. Subfascial drainage was also implemented. The patient's postoperative recovery was smooth, and she was discharged on the fourth postoperative day. Histopathological examination confirmed the complete tumor removal with clear margins and also ruled out malignancy.

## Discussion

This case presentation highlights the complexities surrounding the diagnosis and management of CBTs. The large size (8.5 cm) and 13-year history of our case are unusual and exemplify the significant challenges of a late presentation. While often presenting as an incidental finding due to their slow growth and painless nature, the underlying anatomical location and potential functional significance demand careful consideration. The Shamblin I stage is easy to resect, while II and III can pose further challenges.

This patient's tumor, classified as Shamblin type III due to the complete encasement of the carotid bifurcation, shows the challenges associated with these highly vascular neoplasms. The increased surgical difficulty and risk of morbidity and mortality compared to lower Shamblin classifications underscore the importance of accurate early preoperative diagnosis and meticulous surgical planning [10].

The importance of a multifaceted approach to diagnosis is represented in this case. While routine laboratory investigations provided an initial baseline and ruled out thyroid or other blood-related disorders, imaging techniques like CT angiography and MRI played a crucial role in confirming the tumor's size, location, and relationship to the carotid vessels [11]. The characteristic "Lyre sign" on angiography, visualizing the splaying of internal and external carotid arteries, helps with definitive diagnosis and also aids in surgical planning.

The emphasis on surgical resection as the definitive treatment expands on the limitations of alternative modalities. Radiotherapy and embolization, which are intended to decrease tumor size preoperatively, lack a curative potential. The contraindications for biopsy due to the highly vascular nature of CBTs further highlight the reliance on non-invasive diagnostic techniques alone [12].

CBT surgery comes with a high risk of complications such as perioperative stroke (4%) and cranial nerve injury (17%), particularly more prevalent in Shamblin III [13]. Modern techniques and detailed planning in minimizing complications can help improve outcomes. Newer approaches to reduce tumor size and utilize advanced energy sources for precise dissection during surgery contribute to better results [14].

## Conclusions

This case confirms the importance of considering a broad differential diagnosis for painless neck masses, even in the absence of obvious symptoms. The accurate diagnosis of a complex Shamblin III CBT highlights the crucial role of imaging techniques during preoperative planning and the inherent challenges associated with the resection of highly vascular lesions. Although it is considered high risk, a well-planned surgery remains the definitive treatment in managing these challenging tumors. This case underscores that for even complex Shamblin III CBTs, meticulous preoperative planning and precise sub-adventitial dissection can achieve complete resection with the preservation of neurological function and an excellent outcome.
